# Cerebellar dysfunction in glaucoma patients

**DOI:** 10.1093/braincomms/fcaf401

**Published:** 2025-10-15

**Authors:** Anisha Kasi, Ji Won Bang, Vivek Trivedi, Jeannie M Au, Ian P Conner, Gadi Wollstein, Joel S Schuman, Rakie Cham, Kevin C Chan

**Affiliations:** New York University Grossman School of Medicine, NYU Langone Health, New York University, New York, NY 10017, USA; Icahn School of Medicine at Mount Sinai, New York, NY 10029, USA; New York University Grossman School of Medicine, NYU Langone Health, New York University, New York, NY 10017, USA; Spencer Center for Vision Research, Byers Eye Institute, Department of Ophthalmology, Stanford University School of Medicine, Palo Alto, CA 94303, USA; New York University Grossman School of Medicine, NYU Langone Health, New York University, New York, NY 10017, USA; New York University Grossman School of Medicine, NYU Langone Health, New York University, New York, NY 10017, USA; Department of Ophthalmology, School of Medicine, University of Pittsburgh, Pittsburgh, PA 15213, USA; Department of Bioengineering, Swanson School of Engineering, University of Pittsburgh, Pittsburgh, PA 15213, USA; Wills Eye Hospital, Glaucoma Service and Vickie and Jack Farber Vision Research Center, Philadelphia, PA 19107, USA; Sidney Kimmel Medical College of Thomas Jefferson University, Philadelphia, PA 19107, USA; Wills Eye Hospital, Glaucoma Service and Vickie and Jack Farber Vision Research Center, Philadelphia, PA 19107, USA; Sidney Kimmel Medical College of Thomas Jefferson University, Philadelphia, PA 19107, USA; Drexel University School of Biomedical Engineering, Science and Health Studies, Philadelphia, PA 19104, USA; Department of Ophthalmology, School of Medicine, University of Pittsburgh, Pittsburgh, PA 15213, USA; Department of Bioengineering, Swanson School of Engineering, University of Pittsburgh, Pittsburgh, PA 15213, USA; New York University Grossman School of Medicine, NYU Langone Health, New York University, New York, NY 10017, USA; Spencer Center for Vision Research, Byers Eye Institute, Department of Ophthalmology, Stanford University School of Medicine, Palo Alto, CA 94303, USA; Department of Ophthalmology, School of Medicine, University of Pittsburgh, Pittsburgh, PA 15213, USA; Department of Bioengineering, Swanson School of Engineering, University of Pittsburgh, Pittsburgh, PA 15213, USA

**Keywords:** brain, cerebellum, functional connectivity, glaucoma, magnetic resonance imaging

## Abstract

Glaucoma patients often have higher injurious fall rates compared to healthy older adults. However, little is known about the underlying neural mechanisms. Recent evidence shows cerebral changes beyond the visual pathway of glaucoma patients, yet it remains unclear whether the cerebellum, which plays an important role in balance and motor control, is involved in glaucoma. In this study, we sought to investigate cerebellar functional connectivity changes in glaucoma by comparing 32 glaucoma subjects and 10 age-matched healthy control subjects who underwent resting-state functional magnetic resonance imaging at 3 Tesla with eyes closed. After conducting both regions-of-interest and seed-to-voxel analyses, we found that the functional connectivity within the cerebellum tended to be weakened in glaucoma patients compared to healthy controls, whereas the functional connectivity between some cerebral and cerebellar regions showed opposite changes in the same glaucoma subjects. Our findings underscore the potential role of cerebellar and cerebro-cerebellar dysfunction in postural and cognitive control in glaucoma patients. Taken together, these observations implicate the widespread brain changes in glaucoma beyond the cerebral regions into the cerebellum that may underlie the neural underpinnings of impaired balance control in this disease.

## Teaser

Kasi *et al*. report that glaucoma patients possessed weakened functional connectivity within the cerebellum compared to healthy controls, whereas that between some cerebral and cerebellar regions showed opposite changes in the same subjects. These observations implicate the widespread brain changes that may underpin the impaired balance control in glaucoma.

## Graphical Abstract

**Graphical Abstract:**
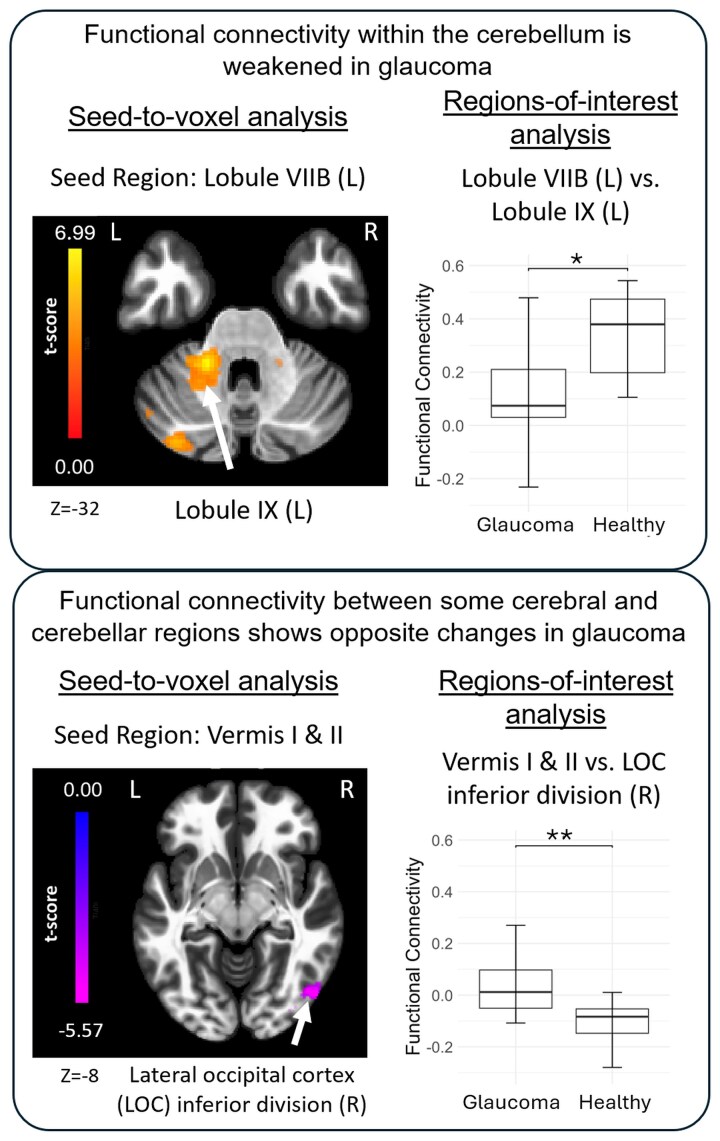

## Introduction

Over one-third of older adults aged 65 or older fall each year, which can cause severe injuries and loss of mobility.^1^ Among them, patients with glaucoma, a neurodegenerative disease characterized by progressive loss of retinal ganglion cells and irreversible vision loss, are over three times more likely to fall compared to healthy individuals.^2^ Glaucoma patients with greater visual impairment are found to experience falls more frequently.^3^ However, even with their eyes closed, glaucoma patients tend to fall more easily, indicating that visual impairment may not be the only factor contributing to falls.^4^ Recent neuroimaging research has demonstrated widespread brain changes both within the visual system and beyond in glaucoma.^5,6^ Indeed, glaucoma patients may exhibit impaired ability to process integration of visual, auditory, somatosensory, and vestibular information.^4^ Thus, it has been suggested that glaucoma may affect areas of the brain involved in multisensory integration for balance control.^7^

While many studies have focused on the cerebral changes in glaucoma patients, less is known about the involvement of the cerebellum in glaucoma, despite its function in coordinating a wide range of motor and cognitive processes. Recent studies have found alterations of the cerebellum in glaucoma, including lower grey matter volume of the cerebellum in primary open-angle glaucoma (POAG) patients with correlation to lower retinal nerve fibre layer thickness,^8^ and decreased functional connectivity (FC) between primary visual cortex and anterior lobe of the left cerebellum in POAG.^9^ In contrast, another study seeking to explore whole-brain connectivity in glaucoma patients included the broader cerebellar network as part of this analysis and did not find altered FC in the cerebellar network in POAG or normal-tension glaucoma patients.^5^ Given the mixed literature regarding cerebellar changes in glaucoma, we sought to examine these changes further by studying FC changes in the cerebellum at the regional level.

Different cerebellar regions have been found to participate in distinct functional networks, such as the lobules II-VI and VIIB in the sensorimotor networks, VI-VIII and IX-X in the oculomotor networks, and IX-X in the vestibular networks.^10^ These relevant functions necessitate further examination of the connectivity not only within the cerebellum but also between the cerebellum and cerebrum in glaucoma patients. The interactions between cerebellar and cerebral nuclei may underlie vestibular integration during self-motion,^11^ suggestive of the importance of studying cerebellar connectivity with other brain areas in glaucoma patients to better unveil the pathogenesis behind glaucoma-related non-visual impairments.

In this study, we used resting-state functional magnetic resonance imaging (MRI) to non-invasively investigate the FC changes within cerebellar regions and between cerebellar and cerebral regions in glaucoma. Our findings can offer insight into the underlying neural mechanisms behind motor and cognitive impairments of glaucoma patients, in order to guide more effective multimodal interventions beyond lowering intraocular pressure and vision preservation in glaucoma.

## Materials and methods

This study was approved by the institutional review board at the University of Pittsburgh. It adhered to the tenets of the Declaration of Helsinki. Informed consent was obtained from all subjects prior to participation. Thirty-two glaucoma subjects (age = 62.5 ± 8.5 years; mean ± standard deviation; 40.6% male) and 10 healthy control subjects (age = 64.5 ± 9.2 years; 30.0% male) underwent anatomical MRI and resting-state task-free functional MRI. The average retinal nerve fibre layer thickness was 74.6 ± 13.0 µm and 74.5 ± 13.0 µm in the left and right eyes of glaucoma subjects, respectively, and 89.0 ± 9.8 µm and 88.6 ± 11.1 µm in the left and right eyes of in healthy controls, respectively (two-tailed unpaired t-tests: *P* < 0.05 between glaucoma and healthy subjects in either eye). The visual field mean deviation was −4.37 ± 4.92 dB and −3.80 ± 6.17 dB in the left and right eyes of glaucoma subjects, respectively, and −0.64 ± 0.57 dB and −1.17 ± 0.92 dB in the left and right eyes of in healthy controls, respectively (two-tailed unpaired t-tests: *P* < 0.05 between glaucoma and healthy subjects in the left eye only). Among the 32 glaucoma subjects, 29 subjects had open-angle glaucoma and 3 had angle-closure glaucoma. All participants had a best corrected visual acuity of 20/60 or better and had no present indications of neurological disorders. Individuals with any additional retinal disorders were excluded from participation in the study.

### MRI protocols

All MRI experiments were acquired using a 3-Tesla Allegra head scanner (Siemens, Erlangen, Germany) with a single-channel transmit-receive birdcage head coil. Conventional anatomical MRI was performed covering the whole brain using a 3D magnetization-prepared rapid acquisition with gradient echo (MPRAGE) pulse sequence with the following parameters: repetition time (TR) = 1.4 s, echo time (TE) = 2.5 ms, inversion time (TI) = 800 ms, field of view = 25.6 × 25.6 × 17.6 cm^3^, 256 × 256 imaging matrix in-plane, flip angle = 8°, and 176 contagious sagittal slices. For resting-state functional MRI, blood-oxygenation-level-dependent (BOLD) images were acquired using a single-shot echo-planar-imaging (EPI) pulse sequence with TR = 2 s, TE = 26 ms, field-of-view = 20.5 × 20.5 cm^2^, 104 × 104 imaging matrix, and 28 contiguous 3 mm thick axial slices. The resting-state functional MRI session was 8 min in duration with eyes closed.

### Statistical analysis

The Functional Connectivity (CONN) and Statistical Parametric Mapping (SPM) toolboxes were used for data preprocessing and regions-of-interest (ROI) analyses of resting-state functional MRI. We designated the ROIs by centring 10 mm spheres at coordinates specified in the Automated Anatomical Labeling and the Harvard-Oxford Atlas. The exact ROIs used are detailed in Supplementary Table 1. Of the 28 available cerebellar ROIs (Regions 31–32 and 139–164, Supplementary Table 1) in the Automated Anatomical Labeling and the Harvard-Oxford Atlas, we only included 26 non-overlapping cerebellar ROIs (Regions 139–164, Supplementary Table 1) as the distinct seed regions. FC was measured between each cerebellar seed region and 164 ROIs within the cerebellum and cerebrum. The results of this ROI-to-ROI analysis yielded a subset of cerebellar seed regions that displayed altered FC in glaucoma patients compared to healthy controls. We then used the brain regions with significantly altered FC in the ROI-to-ROI analysis as the seeds for a secondary seed-to-voxel analysis. To adjust for multiple comparisons, false discovery rate (FDR) corrections were applied in both ROI-to-ROI and seed-to-voxel analyses. Results were considered significant when the FDR-corrected P-values (p-FDR) < 0.05. Details of the processing conducted in CONN can be found in Supplementary Methods.

For statistical analysis, the ROI-to-ROI analysis used two-sample *t*-tests to compare FC of brain ROI pairs between glaucoma and healthy subjects. The seed-to-voxel analysis used *post-hoc t*-tests to compare FC of the seed brain ROI and clusters of voxels between glaucoma and healthy subjects. Both ROI-to-ROI and seed-to-voxel analyses were adjusted for age and sex.

## Results

### ROI-to-ROI analysis

**Figure 1:**
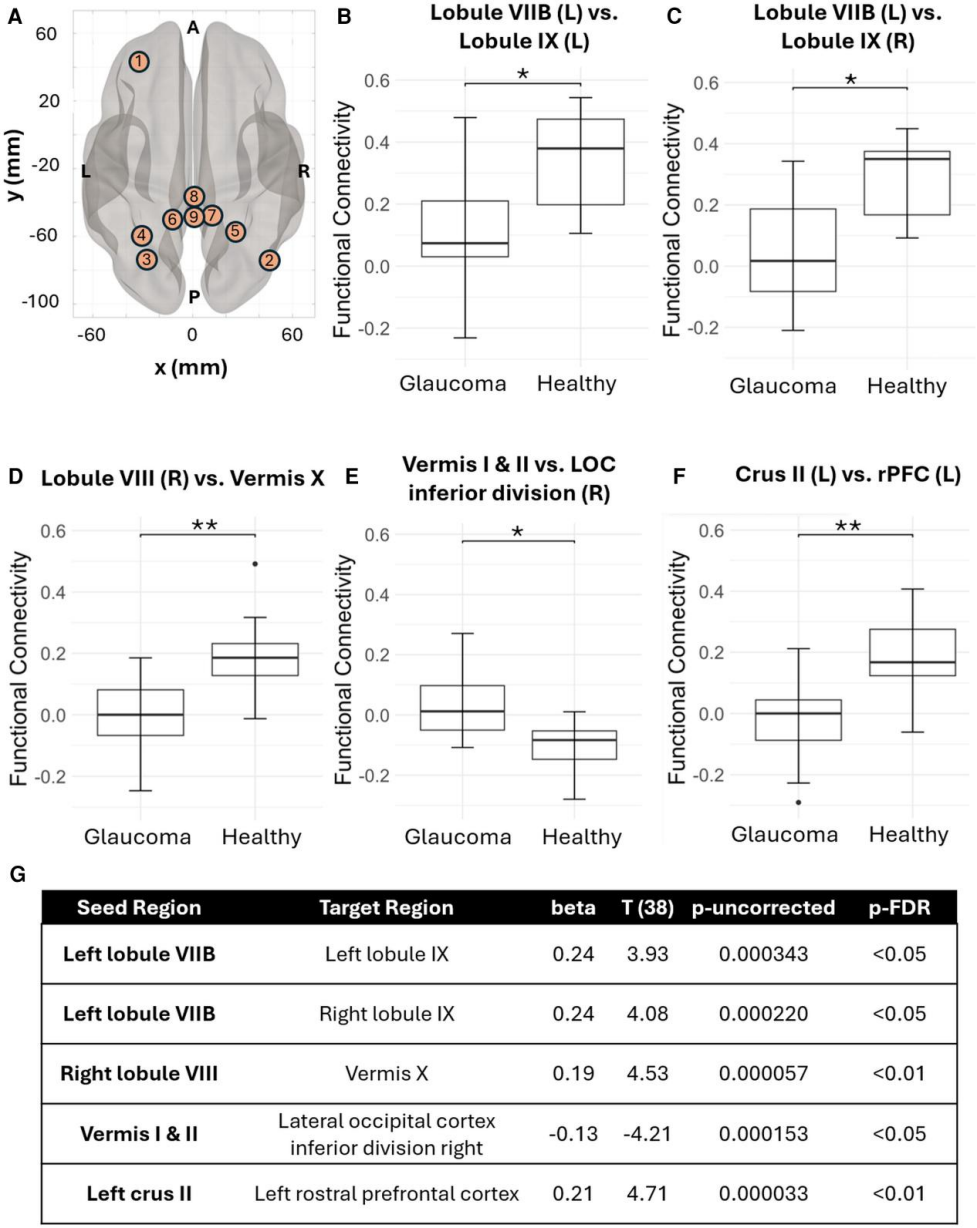
**ROI-to-ROI based differences in functional connectivity between glaucoma and healthy groups (A)** shows brain regions-of-interest (ROIs) that exhibited significance in functional connectivity (FC) differences between glaucoma and healthy subjects using ROI-to-ROI analysis. Anatomical orientations are labelled as follows: L for left, R for right, P for posterior, and A for anterior. The relevant brain regions include: (1) left rostral prefrontal cortex (rPFC), (2) right lateral occipital cortex (LOC) inferior division, (3) left crus II, (4) left lobule VIIB, (5) right lobule VIII, (6) left lobule IX, (7) right lobule IX, (8) vermis I & II, and (9) vermis X. Directions are indicated on the axes, with the coordinates displayed in the MNI space. **(B) through (F)** show box-and-whisker plots of these differences between glaucoma and healthy subjects, with **(B)** left lobule VIIB versus left lobule IX, **(C)** left lobule VIIB versus right lobule IX, **(D)** right lobule VIII versus vermis X, **(E)** vermis I & II versus right LOC inferior division, and **(F)** left crus II versus left rPFC. In descending order, points represent: maximum, third quartile, median, first quartile, and minimum. Circle dots represent outliers. Significance of a two-sample *t*-test (*N*_glaucoma_ = 32, *N*_healthy_ = 10) is indicated above each plot, with *p-FDR < 0.05; **p-FDR < 0.01. **(G)** shows a table of the results of the ROI-to-ROI analysis, including the seed region, target region, beta value, *t*-statistic, uncorrected *P*-value, and the corrected p-FDR value.

Using 26 distinct cerebellar regions as seed ROIs, 5 ROI pairs were found to exhibit significantly different FC between glaucoma and healthy subjects. These ROI pairs include the left lobule VIIB and left lobule IX, left lobule VIIB and right lobule IX, right lobule VIII and vermis X, and vermis I and II and the right inferior division of the lateral occipital cortex (LOC), and left cerebellar crus II and left rostral prefrontal cortex (rPFC), (Fig. 1A-F). Except for the significantly higher FC between vermis I and II and the right inferior division of the LOC in glaucoma, all the other 4 ROI pairs showed a significantly lower FC in glaucoma subjects as compared to healthy subjects. Figure 1G lists the corresponding beta value, *t*-statistic, uncorrected *P* values, and p-FDR for each of the significant ROI pairs. Supplementary Fig. 1 shows the heatmap of the group differences in FC between the 26 cerebellar seed ROIs and all 164 cerebral and cerebellar ROIs.

### Seed-to-voxel analysis

**Figure 2:**
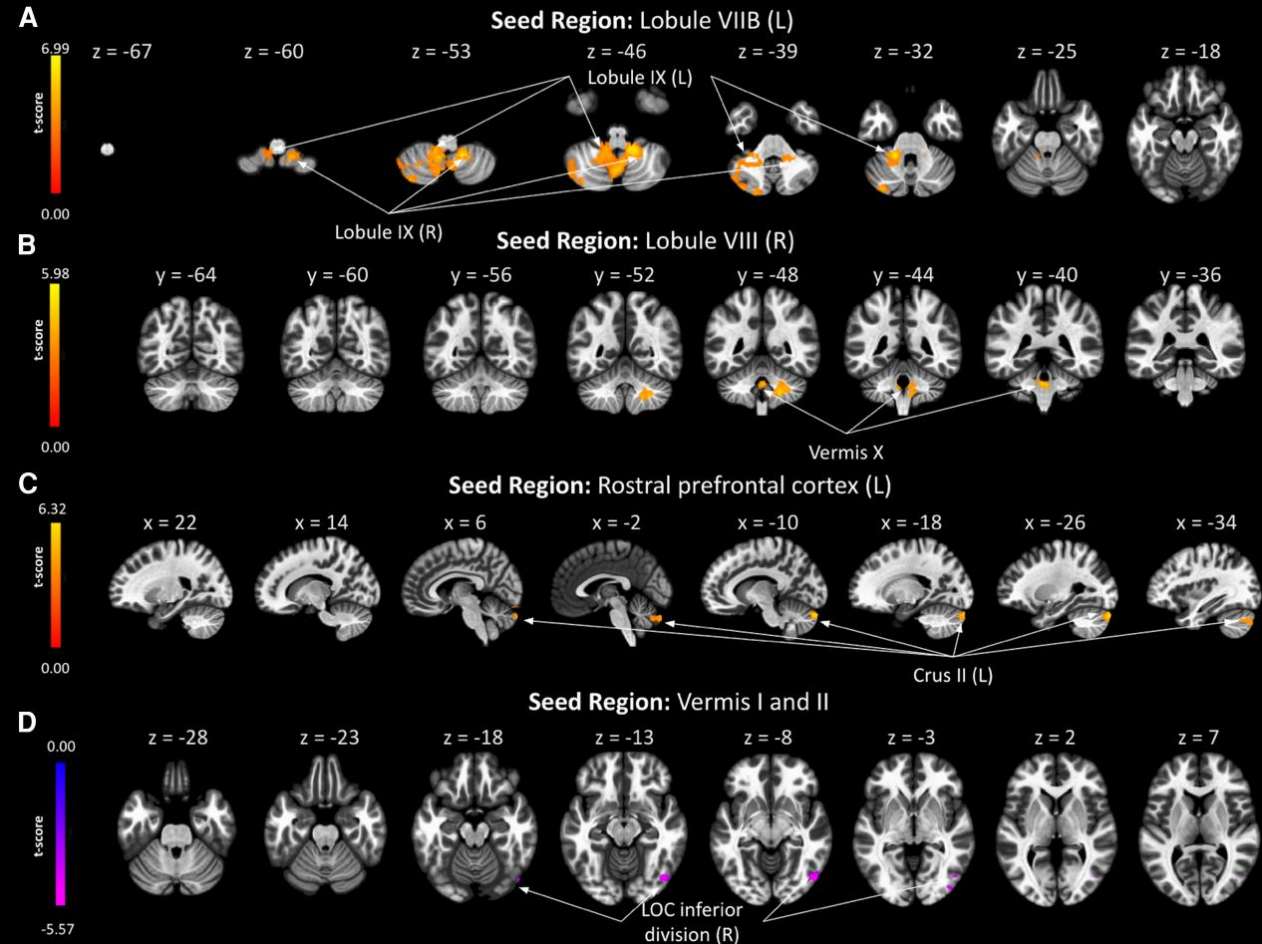
**Seed-to-voxel based differences in functional connectivity between glaucoma and healthy groups.** The colour bar on the left of each panel represents the *t*-statistic for a *post-hoc t*-test (*N*_glaucoma_ = 32, *N*_healthy_ = 10) of the functional connectivity (FC) differences between glaucoma and healthy subjects. Left is labelled as (**L**) and right is labelled as (R). Positive *t*-statistics indicate that FC is lower in the glaucoma group than the healthy group, while negative *t*-statistics indicate that FC is greater in the glaucoma group than the healthy group. The brain slices in each panel represent the *t*-score voxel maps overlaid on the axial (**A**) and (**D**), coronal (**B**), or sagittal (**C**) anatomical images at their corresponding *z*-, *y*-, or *x*-MNI coordinates using different seed regions. Only voxels exceeding the statistical threshold of p-FDR < 0.05 are displayed on the *t*-score maps. **(A)** Using the left lobule VIIB as the seed region, clusters with significant groupwise differences in FC were observed in the left and right lobule IX. **(B)** Using the right lobule VIII as the seed region, clusters with significant groupwise differences in FC were observed in the vermis X. **(C)** Using the left rostral prefrontal cortex (rPFC) as the seed region, clusters with significant groupwise differences in FC were observed in the left crus II. **(D)** Using the vermis I and II as the seed region, clusters with significant groupwise differences in FC were observed in the right inferior division of the lateral occipital cortex (LOC).

Having found 5 ROI pairs, including 9 regions that exhibited significantly different FC between glaucoma and healthy subjects by ROI-to-ROI analysis, we further examined whether similar patterns existed at the voxel level. Using these 9 regions (left lobule VIIB, right lobule VIII, left lobule IX, right lobule IX, vermis I and II, vermis X, left crus II, left rPFC, and right inferior division of the LOC) as our seed regions, we identified in Tables 1 and 2, the corresponding significant cluster of voxels, along with the regions included in the cluster, the cluster’s peak Montreal Neurological Institute (MNI) coordinates, the *t*-statistic for a *post-hoc t*-test between glaucoma and healthy subjects, the cluster size, and p-FDR. All 5 ROI pairs found to be significant in the ROI-to-ROI analysis also exhibited significant group FC differences in this seed-to-voxel analysis, as highlighted in Tables 1 and 2 and Fig. 2. In addition, some new brain regions in the cerebrum, cerebellum, and brainstem were identified to be FC altered with the 9 seed ROIs in glaucoma (Tables 1 and 2).

**Table 1:** Results of seed-to-voxel functional connectivity (FC) analysis in glaucoma versus healthy sighted controls using cerebellar lobules as seeds

Seed	Regions	Peak MNI Coordinate			*t*-Statistic	Cluster size	p-FDR
Lobule VIIB (L)	Crus II (L), **Lobule IX (L),** **Lobule IX (R)**	14	−50	−52	6.99	3594	<0.000001
	Lobule VIIB (R), Lobule VIII (R), Crus II (R)	26	−80	−56	4.87	538	0.000074
	LOC superior division (R), Cuneal cortex (R)	26	−70	26	−4.50	194	0.036869
Lobule VIII (R)	Lobule VIII (L), Lobule VIIB (L)	−32	−74	−60	5.22	498	0.000102
	Lobule IX (R), Lobule VIII (R), Brainstem, **Vermis X**	−02	−42	−34	5.98	532	0.000102
	Crus II (L)	−8	−84	−50	4.46	217	0.016990
Lobule IX (L)	Crus II (L), Crus I (L), **Lobule VIIB (L)**	−36	−78	−42	5.74	1177	<0.000001
	Crus II (R), Crus I (R)	42	−62	−42	5.69	450	0.000016
	Postcentral gyrus (L), Precentral gyrus (L)	−32	−34	66	−5.55	310	0.000323
	Brainstem, Lobules IV and V (L), Lobule III (L)	−10	−34	−42	5.15	276	0.000594
	Angular gyrus (R), Supramarginal gyrus posterior division (R), LOC superior division (R)	48	−40	44	4.30	195	0.004750
Lobule IX (R)	**Lobule VIIB (L)**, Lobule VIII (L), Crus II (L)	−34	−68	−54	5.41	480	0.000801

Bolded and underlined regional pairs also showed group FC differences in the ROI-to-ROI analysis in Fig. 1G. Positive *t*-statistics indicate that the FC is lower in the glaucoma group than the healthy group, while negative *t*-statistics indicate that the FC is greater in the glaucoma group than the healthy group. Left is labelled as (L) and right is labelled as (R).

**Table 2:** Results of seed-to-voxel functional connectivity (FC) analysis in glaucoma versus healthy sighted controls using other cerebellar and cerebral seeds

Seed	Regions	Peak MNI coordinate			*t*-statistic	Cluster size	p-FDR
Vermis I & II	**LOC inferior division (R)**	50	−70	−8	−5.57	226	0.048530
Vermis X	Brainstem, Lobule IX (R), Lobule X (R)	10	−36	−52	5.91	366	0.000349
	Crus II (L), Lobule VIII (L), Lobule VIIB (L)	−6	−78	−46	5.95	496	0.000047
	Frontal pole (R)	38	56	4	5.53	191	0.015889
	**Lobule VIII (R)**, Crus II (R)	28	−56	−62	6.12	142	0.048089
Crus II (L)	Crus II (L), Lobule IX (L), Lobule IX (R)	−10	−52	−28	6.50	1896	<0.000001
	LOC superior division (L), Occipital pole (L)	−32	−90	24	−4.39	549	0.000191
	LOC superior division (R)	22	−70	44	−5.12	290	0.010180
Rostral prefrontal cortex (rPFC) (L)	**Crus II (L),** Crus I (L)	2	−88	−32	6.32	1257	<0.000001
	LOC superior division (R)	34	−68	44	4.28	279	0.009073
	Precentral gyrus (L), Middle frontal gyrus (L)	−40	8	28	4.87	190	0.040166
Lateral Occipital Cortex (LOC), inferior division (R)	Lingual gyrus (R), Occipital fusiform gyrus (R), Intracalcarine cortex (R)	18	−82	−8	4.64	294	0.010120
	Lobule IX (L), Lobule VIII (L), Vermis IX	−10	−48	−30	−5.01	198	0.040709

Bolded and underlined regional pairs also showed group FC differences in the ROI-to-ROI analysis in Fig. 1G. Positive *t*-statistics indicate that the FC is lower in the glaucoma group than the healthy group, while negative *t*-statistics indicate that the FC is greater in the glaucoma group than the healthy group. Left is labelled as (L) and right is labelled as (R).

## Discussion

This research addressed an underexplored aspect of glaucoma by linking cerebellar and cerebrocerebellar dysfunction to postural and cognitive impairments, offering potential insights into non-visual interventions. Specifically, our study sought to build on past findings that indicated cerebellar structural and functional changes at the network level in glaucoma^8,9^ by more precisely comparing FC between cerebellar subregions. Despite initial evidence suggesting local changes in the regional homogeneity and degree of centrality in the cerebellar cortex of glaucoma patients,^12-14^ how different cerebellar regions coordinate with one another and with the rest of the brain in glaucoma remains largely unclear.^8^ In this resting-state functional MRI experiment, our findings indicated significantly lower FC within the cerebellum of glaucoma patients compared to healthy controls, whereas the FC between some cerebral and cerebellar regions showed opposite changes in the same glaucoma subjects. These observations implicate the involvement of brain changes in glaucoma beyond the cerebral regions into the cerebellum and may underlie the neural mechanisms of impaired balance control in glaucoma.

In this study, the bilateral lobule IX exhibited weakened FC with the left lobule VIIB in glaucoma patients as compared to healthy controls. The lobule IX, also known as the cerebellar tonsil, is a key player in the head-to-world reference frame conversion, receiving vestibular and proprioceptive signals in relation to body and head positions,^15^ whereas lobule VIIB, also known as the inferior semilunar lobule, is involved in higher motor planning.^16^ Left lobule VIIB and bilateral lobule IX have previously been implicated in eye movements, such as gaze-evoked nystagmus^17^ and saccades,^18^ with abnormal activity in these two cerebellar subregions associating with greater saccadic errors.^19^ Furthermore, in a recent study of 43 patients with cerebellar strokes, saccadic dysmetria was observed among 69.8% of patients.^20^ Among these cerebellar stroke patients, patients with posterior inferior cerebellar artery infarctions, particularly affecting cerebellar lobules VIIB and IX, displayed ipsiversive saccadic dysmetria, underscoring the importance of the cerebellar tonsil and inferior semilunar lobule in saccades.^20^ Interestingly, recent studies have shown that saccades may be impacted in glaucoma patients.^21,22^ Thepass *et al.* found that saccadic reaction times were significantly slower in glaucoma patients, with reaction time increasing with disease severity.^21^ Saccadic reaction times slowed even in normal areas of the visual field where glaucomatous loss had not yet occurred, suggesting that saccades may reflect a higher-order brain change in glaucoma that precedes visual field loss. Furthermore, Yeon *et al.* recently found that saccadic trajectories are affected in glaucoma in terms of increased saccadic reaction time, departure angle, and arrival angle for 14° vertical visual, as compared to healthy controls.^22^ In terms of brain involvements, using Granger causality analysis, Zhong *et al.* found that as intraocular pressure increases with glaucoma severity, brain atrophy occurs first in the cerebellum, visual cortex, and frontal eye fields (i.e. regions that control oculomotor function), followed by extensions to the parietal and temporal lobes.^23^ Given the potential role of lobules VIIB and IX in saccades,^19^ the weakened FC between these cerebellar regions may implicate the underlying neural mechanisms of glaucomatous changes in saccades.

Furthermore, it has been proposed that lobule IX is a multimodal area that interconnects three pathways, namely the dorsal attentional network, which encompasses lobule VIIB and caudal lobule IX, with the executive control network and the default-mode network, both of which include lobule IX.^24^ Together, these three pathways may be important for visuospatial processing. Thus, the weakened FC that we observed between lobule VIIB and lobule IX may form the neural basis for impaired visuomotor integration in glaucoma.

Other members of the default-mode network, crus II and rPFC, were also implicated in our study. Crus II was found to exhibit a significantly lower FC with rPFC in glaucoma patients compared to healthy controls. The default-mode network functions in tasks that require focused attention, such as visuospatial tasks, and has been implicated in glaucoma previously.^25,26^ In a recent study of 34 patients with primary angle-closure glaucoma, patients exhibited weakened FC within the default-mode network, suggesting a neurological mechanism associated with visual and cognitive dysfunction in glaucoma.^25^ Likewise, in a sample of 25 patients with POAG, the default-mode network was found to have a decreased connection with the visual network relative to normal controls, which positively correlated with glaucoma severity as assessed by visual field mean deviation.^26^ Furthermore, Shu *et al.* recently used bidirectional Mendelian randomization analysis between glaucoma and brain functional networks, and suggested that increased risk of glaucoma leads to brain functional network abnormalities in the subcortical cerebellar network and the default-mode network.^27^ Thus, the weakened FC between crus II and rPFC observed in our study may reflect disruptions of the default-mode network in glaucoma pathogenesis.

We also found that the right lobule VIII in glaucoma patients had weakened FC with vermis X compared to healthy controls. Lobule VIII, also known as the biventral lobule, receives inputs from the primary motor cortex and contains somatomotor representations of the hand and individual digits.^28^ Interestingly, decreased grey matter volume of lobule VIII has been observed in other neurodegenerative diseases, including certain subtypes of Parkinson’s disease and schizophrenia, which may link to the motor deficits seen in these diseases.^29,30^ Cerebellar vermis X, also known as the nodulus, is responsible for integrating vestibular input from the otolith organs and semicircular canals with proprioceptive input and visual motion information in order to compute the brain’s representation of our self-motion relative to gravity.^31^ Our finding of the lower FC between right lobule VIII and vermis X may underlie a disruption of normal integration of spatial processing and self-motion in glaucoma patients.

In contrast to the lower FC in the brain regions aforementioned, the vermis I and II and the right inferior division of the LOC were found to have a significantly higher FC in glaucoma patients. Vermis I and II, also known as the lingula, are part of the anterior vermis, which is responsible for transforming vestibular signals into a body-centred reference frame for control of posture, balance, and gait.^31^ Given that the LOC plays an important role in shape perception and object recognition,^32^ the increased FC between vermis I and II and the LOC may reflect a compensatory effect following visual deficits in glaucoma patients, which could then improve interactions with the internal (e.g. posture) and external (e.g. balance and object recognition) worlds.

Using the seed-to-voxel analysis, we found additional areas with altered FC in glaucoma (Tables 1 and 2). Among these brain regions, the cerebro-cerebellar connections typically showed increased FC in glaucoma patients, including vermis I & II versus right LOC inferior division, crus II versus left and right LOC superior division, left lobule VIIB versus right LOC superior division and right cuneal cortex, and left lobule IX versus pre-central and post-central gyrus, among other pairs. Further studies are warranted that determine if this reflects a compensatory increase in cerebral regions involved in balance and object recognition following visual deficits in glaucoma. In particular, amongst the proposed mechanisms of falls in glaucoma, one key candidate is the reduction in peripheral vision, hindering the ability to detect environmental hazards. However, the findings of this study related to the cerebellar dysfunction in glaucoma suggest this may not be the only fall mechanism in this population. It is well established that the cerebellum plays a key role in the central integration of multisensory inputs relevant for balance, namely visual, vestibular, and somatosensory information.^33-35^ Effective integration of these sensory channels, a process often termed multisensory re-weighting or integration, involves (1) resolving conflicting sensory inputs if two or more channels provide contradictory body state-related information and (2) relying on accurate sensory channels to generate postural adjustments if one or more afferent inputs are inaccurate, noisy or absent.^36^ Given that peripheral vision is often reduced in patients with glaucoma, the ability to effectively perform sensory re-weighting, potentially via neuroadaptation of the cerebro-cerebellar connections, becomes important to maintaining balance.

Our study is limited by the small sample size, especially for the healthy control group. Despite this limitation, the methods included appropriate multiple-comparison corrections for the imaging data and still the results showed several FC differences in glaucoma patients consistently via the dual ROI-to-ROI and seed-to-voxel analyses, which reflected methodological rigour. The medication use may also confound the observed FC differences. Future studies should consider not only FC but also structural connectivity and behavioural assessments with larger samples across different disease severity and types before or after medication use, in order to improve the generalizability pertaining to cerebellar changes and their structure−function−behaviour relationships in glaucoma.

The findings of this study are clinically important for two reasons: (1) measuring and unveiling the impact of glaucoma on central sensory processing systems in the postural control domain will aid in the assessment and management of patients with glaucoma. Specifically, this information can identify who is at a greater risk of experiencing a fall; (2) the research will also lead to more effective balance therapy and education for the patient. Currently, physical therapists who treat patients with balance disorders use different approaches depending upon the amount of central versus peripheral involvement. Central sensory integration impairments require differing therapeutic techniques,^37,38^ and the same would apply to patients with glaucoma. Different occupational therapy approaches may also be developed to target the key factors causing falls in patients with glaucoma.^39^ In summary, an increased understanding of the central sensory integration system would inform therapists on the best approach for their patients with glaucoma.

## Conclusion

Our study showed evidence of altered FC within the cerebellum and between the cerebellum and cerebrum in glaucoma patients compared to healthy controls. These findings may help in the development of multimodal interventions to address the burden of glaucoma on basic activities like balancing, standing, and walking. Future studies should examine the pathogenesis behind these weakened cerebellar connectivity and bidirectional cerebro-cerebellar connectivity changes in glaucoma and their respective effects on visuomotor function (e.g. saccades) and behaviour (e.g. postural and balance control; cognitive tasks) in glaucoma patients.

## Supplementary Material

fcaf401_Supplementary_Data

## Data Availability

The datasets collected and analysed during the current study are available from the corresponding author on reasonable request.
