# Supplementary material for: Cerebellar dysfunction in glaucoma patients
Source: Brain Commun. 2025 Oct 15;7(6):fcaf401. doi: 10.1093/braincomms/fcaf401 (PMC12579274; doi:10.1093/braincomms/fcaf401)
Supplement: fcaf401_Supplementary_Data [file fcaf401_supplementary_data.pdf]

SUPPLEMENTARY MATERIAL

Title: Cerebellar dysfunction in glaucoma patients

Author List: Anisha Kasi, Ji Won Bang, Vivek Trivedi, Jeannie M. Au, Ian P. Conner, Gadi Wollstein, Joel S. Schuman, Rakie Cham, Kevin C. Chan*

Supplementary Table 1: Regions-of-interest (ROIs) used in functional connectivity MRI analysis

Number	Names of brain regions of interest (ROI)
1	networks.DefaultMode.MPFC
2	networks.DefaultMode.LP (L)
3	networks.DefaultMode.LP (R)
4	networks.DefaultMode.PCC
5	networks.SensoriMotor.Lateral (L)
6	networks.SensoriMotor.Lateral (R)
7	networks.SensoriMotor.Superior
8	networks.Visual.Medial
9	networks.Visual.Occipital
10	networks.Visual.Lateral (L)
11	networks.Visual.Lateral (R)
12	networks.Salience.ACC
13	networks.Salience.AInsula (L)
14	networks.Salience.AInsula (R)
15	networks.Salience.RPFC (L)
16	networks.Salience.RPFC (R)
17	networks.Salience.SMG (L)
18	networks.Salience.SMG (R)
19	networks.DorsalAttention.FEF (L)
20	networks.DorsalAttention.FEF (R)
21	networks.DorsalAttention.IPS (L)
22	networks.DorsalAttention.IPS (R)
23	networks.FrontoParietal.LPFC (L)
24	networks.FrontoParietal.PPC (L)
25	networks.FrontoParietal.LPFC (R)
26	networks.FrontoParietal.PPC (R)
27	networks.Language.IFG (L)
28	networks.Language.IFG (R)
29	networks.Language.pSTG (L)
30	networks.Language.pSTG (R)
31	networks.Cerebellar.Anterior
32	networks.Cerebellar.Posterior
33	atlas.FP r (Frontal Pole Right)
34	atlas.FP l (Frontal Pole Left)

35	atlas.IC r (Insular Cortex Right)
36	atlas.IC l (Insular Cortex Left)
37	atlas.SFG r (Superior Frontal Gyrus Right)
38	atlas.SFG l (Superior Frontal Gyrus Left)
39	atlas.MidFG r (Middle Frontal Gyrus Right)
40	atlas.MidFG l (Middle Frontal Gyrus Left)
41	atlas.IFG tri r (Inferior Frontal Gyrus, pars triangularis Right)
42	atlas.IFG tri l (Inferior Frontal Gyrus, pars triangularis Left)
43	atlas.IFG oper r (Inferior Frontal Gyrus, pars opercularis Right)
44	atlas.IFG oper l (Inferior Frontal Gyrus, pars opercularis Left)
45	atlas.PreCG r (Precentral Gyrus Right)
46	atlas.PreCG l (Precentral Gyrus Left)
47	atlas.TP r (Temporal Pole Right)
48	atlas.TP l (Temporal Pole Left)
49	atlas.aSTG r (Superior Temporal Gyrus, anterior division Right)
50	atlas.aSTG l (Superior Temporal Gyrus, anterior division Left)
51	atlas.pSTG r (Superior Temporal Gyrus, posterior division Right)
52	atlas.pSTG l (Superior Temporal Gyrus, posterior division Left)
53	atlas.aMTG r (Middle Temporal Gyrus, anterior division Right)
54	atlas.aMTG l (Middle Temporal Gyrus, anterior division Left)
55	atlas.pMTG r (Middle Temporal Gyrus, posterior division Right)
56	atlas.pMTG l (Middle Temporal Gyrus, posterior division Left)
57	atlas.toMTG r (Middle Temporal Gyrus, temporooccipital part Right)
58	atlas.toMTG l (Middle Temporal Gyrus, temporooccipital part Left)
59	atlas.aITG r (Inferior Temporal Gyrus, anterior division Right)
60	atlas.aITG l (Inferior Temporal Gyrus, anterior division Left)
61	atlas.pITG r (Inferior Temporal Gyrus, posterior division Right)
62	atlas.pITG l (Inferior Temporal Gyrus, posterior division Left)
63	atlas.toITG r (Inferior Temporal Gyrus, temporooccipital part Right)
64	atlas.toITG l (Inferior Temporal Gyrus, temporooccipital part Left)
65	atlas.PostCG r (Postcentral Gyrus Right)
66	atlas.PostCG l (Postcentral Gyrus Left)
67	atlas.SPL r (Superior Parietal Lobule Right)
68	atlas.SPL l (Superior Parietal Lobule Left)
69	atlas.aSMG r (Supramarginal Gyrus, anterior division Right)
70	atlas.aSMG l (Supramarginal Gyrus, anterior division Left)
71	atlas.pSMG r (Supramarginal Gyrus, posterior division Right)
72	atlas.pSMG l (Supramarginal Gyrus, posterior division Left)
73	atlas.AG r (Angular Gyrus Right)
74	atlas.AG l (Angular Gyrus Left)
75	atlas.sLOC r (Lateral Occipital Cortex, superior division Right)
76	atlas.sLOC l (Lateral Occipital Cortex, superior division Left)
77	atlas.iLOC r (Lateral Occipital Cortex, inferior division Right)
78	atlas.iLOC l (Lateral Occipital Cortex, inferior division Left)
79	atlas.ICC r (Intracalcarine Cortex Right)

80	atlas.ICC l (Intracalcarine Cortex Left)
81	atlas.MedFC (Frontal Medial Cortex)
82	atlas.SMA r (Juxtapositional Lobule Cortex -formerly Supplementary Motor Cortex-Right)
83	atlas.SMA L(Juxtapositional Lobule Cortex -formerly Supplementary Motor Cortex-Left)
84	atlas.SubCalC (Subcallosal Cortex)
85	atlas.PaCiG r (Paracingulate Gyrus Right)
86	atlas.PaCiG l (Paracingulate Gyrus Left)
87	atlas.AC (Cingulate Gyrus, anterior division)
88	atlas.PC (Cingulate Gyrus, posterior division)
89	atlas.Precuneous (Precuneous Cortex)
90	atlas.Cuneal r (Cuneal Cortex Right)
91	atlas.Cuneal l (Cuneal Cortex Left)
92	atlas.FOrb r (Frontal Orbital Cortex Right)
93	atlas.FOrb l (Frontal Orbital Cortex Left)
94	atlas.aPaHC r (Parahippocampal Gyrus, anterior division Right)
95	atlas.aPaHC l (Parahippocampal Gyrus, anterior division Left)
96	atlas.pPaHC r (Parahippocampal Gyrus, posterior division Right)
97	atlas.pPaHC l (Parahippocampal Gyrus, posterior division Left)
98	atlas.LG r (Lingual Gyrus Right)
99	atlas.LG l (Lingual Gyrus Left)
100	atlas.aTFusC r (Temporal Fusiform Cortex, anterior division Right)
101	atlas.aTFusC l (Temporal Fusiform Cortex, anterior division Left)
102	atlas.pTFusC r (Temporal Fusiform Cortex, posterior division Right)
103	atlas.pTFusC l (Temporal Fusiform Cortex, posterior division Left)
104	atlas.TOFusC r (Temporal Occipital Fusiform Cortex Right)
105	atlas.TOFusC l (Temporal Occipital Fusiform Cortex Left)
106	atlas.OFusG r (Occipital Fusiform Gyrus Right)
107	atlas.OFusG l (Occipital Fusiform Gyrus Left)
108	atlas.FO r (Frontal Operculum Cortex Right)
109	atlas.FO l (Frontal Operculum Cortex Left)
110	atlas.CO r (Central Opercular Cortex Right)
111	atlas.CO l (Central Opercular Cortex Left)
112	atlas.PO r (Parietal Operculum Cortex Right)
113	atlas.PO l (Parietal Operculum Cortex Left)
114	atlas.PP r (Planum Polare Right)
115	atlas.PP l (Planum Polare Left)
116	atlas.HG r (Heschl"s Gyrus Right)
117	atlas.HG l (Heschl"s Gyrus Left)
118	atlas.PT r (Planum Temporale Right)
119	atlas.PT l (Planum Temporale Left)
120	atlas.SCC r (Supracalcarine Cortex Right)
121	atlas.SCC l (Supracalcarine Cortex Left)
122	atlas.OP r (Occipital Pole Right)

123	atlas.OP l (Occipital Pole Left)
124	atlas.Thalamus r'
125	atlas.Thalamus l'
126	atlas.Caudate r'
127	atlas.Caudate l'
128	atlas.Putamen r'
129	atlas.Putamen l'
130	atlas.Pallidum r'
131	atlas.Pallidum l'
132	atlas.Hippocampus r'
133	atlas.Hippocampus l'
134	atlas.Amygdala r'
135	atlas.Amygdala l'
136	atlas.Accumbens r'
137	atlas.Accumbens l'
138	atlas.Brain-Stem'
139	atlas.Cereb1 l (Cerebellum Crus1 Left)
140	atlas.Cereb1 r (Cerebellum Crus1 Right)
141	atlas.Cereb2 l (Cerebellum Crus2 Left)
142	atlas.Cereb2 r (Cerebellum Crus2 Right)
143	atlas.Cereb3 l (Cerebellum 3 Left)
144	atlas.Cereb3 r (Cerebellum 3 Right)
145	atlas.Cereb45 l (Cerebellum 4 5 Left)
146	atlas.Cereb45 r (Cerebellum 4 5 Right)
147	atlas.Cereb6 l (Cerebellum 6 Left)
148	atlas.Cereb6 r (Cerebellum 6 Right)
149	atlas.Cereb7 l (Cerebellum 7b Left)
150	atlas.Cereb7 r (Cerebellum 7b Right)
151	atlas.Cereb8 l (Cerebellum 8 Left)
152	atlas.Cereb8 r (Cerebellum 8 Right)
153	atlas.Cereb9 l (Cerebellum 9 Left)
154	atlas.Cereb9 r (Cerebellum 9 Right)
155	atlas.Cereb10 l (Cerebellum 10 Left)
156	atlas.Cereb10 r (Cerebellum 10 Right)
157	atlas.Ver12 (Vermis 1 2)
158	atlas.Ver3 (Vermis 3)
159	atlas.Ver45 (Vermis 4 5)
160	atlas.Ver6 (Vermis 6)
161	atlas.Ver7 (Vermis 7)
162	atlas.Ver8 (Vermis 8)
163	atlas.Ver9 (Vermis 9)
164	atlas.Ver10 (Vermis 10)

Supplementary Figure 1:

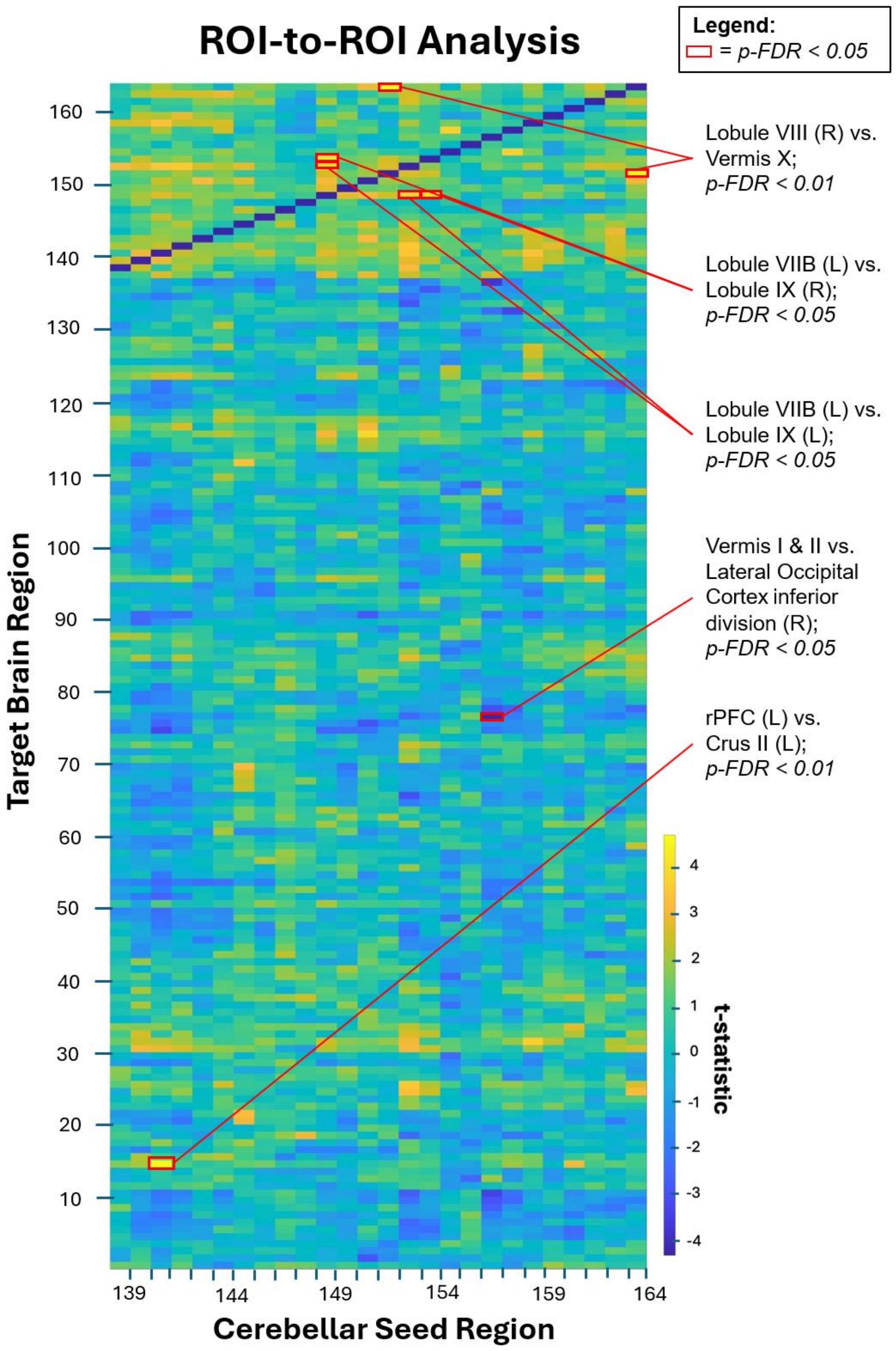

Supplementary Figure 1: Heat maps showing t-statistics for differences in functional connectivity between glaucoma and healthy subjects using ROI-to-ROI analysis with 26 cerebellar ROIs as seeds. The names of the corresponding brain regions in the x-axis and y-axis can be found in Supplementary Table 1. (L) means left-sided and (R) means right-sided. The red box marks ROI pairs in which there is a significant functional connectivity difference [false discovery rate (FDR) corrected p value (p-FDR) <0.05] between the glaucoma subjects and healthy controls in a two-sample t-test ($N_{glaucoma} = 32$, $N_{healthy} = 10$).

Supplementary Methods:

The Functional Connectivity (CONN)[1] (RRID:SCR_009550; release 18.a)[2] and Statistical Parametric Mapping (SPM) (RRID:SCR_007037; release 12.7771)[3] toolboxes were used for data preprocessing and regions-of-interest (ROI) analyses of resting-state functional MRI.

CONN Pipeline:

Preprocessing: Functional and anatomical data were preprocessed using a flexible preprocessing pipeline[4] including realignment with correction of susceptibility distortion interactions, slice timing correction, outlier detection, direct segmentation and MNI-space normalization, and smoothing. Functional data were realigned using SPM realign & unwarp procedure,[5] where all scans were co-registered to a reference image using a least squares approach and a 6-parameter (rigid body) transformation,[6] and resampled using b-spline interpolation to correct for motion and magnetic susceptibility interactions. Temporal misalignment between different slices of the functional data acquired in an interleaved order was corrected following SPM slice-timing correction procedure,[7,8] using sinc temporal interpolation to resample the BOLD timeseries in each slice to a common mid-acquisition time. Potential outlier scans were identified using artifact detection tools[9] as acquisitions with framewise displacement above 0.9 mm or global BOLD signal changes above 5 standard deviations,[10,11] and a reference BOLD image was computed for each subject by averaging all scans excluding outliers. Functional and anatomical data were normalized into standard MNI space, segmented into grey matter, white matter, and cerebrospinal fluid (CSF) tissue classes, and resampled to 2 mm isotropic voxels following a direct normalization procedure[11,12] using SPM unified segmentation and normalization algorithm[13,14] with the default IXI-549 tissue probability map template. Lastly, functional data were smoothed using spatial convolution with a Gaussian kernel of 8 mm full width at half maximum.

Denoising: In addition, functional data were denoised using a standard denoising pipeline[15] including the regression of potential confounding effects characterized by white matter timeseries (5 CompCor noise components), CSF timeseries (5 CompCor noise components), motion parameters and their first order derivatives (12 factors),[16] outlier scans (below 54 factors),[10] session effects and their first order derivatives (2 factors), and linear trends (2 factors) within each functional run, followed by bandpass frequency filtering of the BOLD timeseries[17] between 0.008 Hz and 0.09 Hz. CompCor[18,19] noise components within white matter and CSF were estimated by computing the average BOLD signal as well as the largest principal components orthogonal to the BOLD average, motion parameters, and outlier scans within each subject's eroded segmentation masks. From the number of noise terms included in this denoising strategy, the effective degrees of freedom of the BOLD signal after denoising were estimated to range from 52.5 to 70.2 (average 68.2) across all subjects.[11]

First-level analysis: Seed-based connectivity maps and ROI-to-ROI connectivity matrices were estimated to characterize the patterns of functional connectivity (FC) with 164 ROIs. FC strength was represented by Fisher-transformed bivariate correlation coefficients from a weighted general linear model,[20] defined separately for each pair of seed and target areas, modeling the association between their BOLD signal timeseries.

Group-level analyses were performed using a general linear model.[22] For each individual voxel, a separate general linear model was estimated, with first-level connectivity measures at this voxel as dependent variables (i.e. one independent sample per subject and one measurement per task or experimental condition, if applicable), and groups or other subject-level identifiers as independent variables. Voxel-level hypotheses were evaluated using multivariate parametric statistics with random-effects across subjects and sample covariance estimation across multiple measurements. Inferences were performed at the level of individual clusters (i.e. groups of contiguous voxels). Cluster-level inferences

were based on parametric statistics from Gaussian Random Field theory.[23,24] Results were thresholded using a combination of a cluster-forming p<0.005 voxel-level threshold, and a familywise corrected p-FDR<0.05 cluster-size threshold.[25]

References

1. Whitfield-Gabrieli S, Nieto-Castanon A. Conn: a functional connectivity toolbox for correlated and anticorrelated brain networks. *Brain Connect*. 2012;2(3):125-41. doi:10.1089/brain.2012.0073
2. Nieto-Castanon A, Whitfield-Gabrieli S. CONN functional connectivity toolbox: RRID SCR_009550, release 18. 2018.
3. Penny WD, Friston KJ, Ashburner JT, Kiebel SJ, Nichols TE. Statistical Parametric Mapping: The Analysis of Functional Brain Images. Elsevier; 2011.
4. Nieto-Castanon A. FMRI minimal preprocessing pipeline. *Handbook of functional connectivity Magnetic Resonance Imaging methods in CONN*. Hilbert Press; 2020:3-16.
5. Andersson JL, Hutton C, Ashburner J, Turner R, Friston KJ. Modeling geometric deformations in EPI time series. 2001;13(5):903-919.
6. Friston KJ, Ashburner J, Frith CD, Poline JB, Heather JD, Frackowiak RS. Spatial registration and normalization of images. Human brain mapping; 1995. p. 165-189.
7. Henson RNA, Buechel C, Josephs O, Friston KJ. The slice-timing problem in event-related fMRI. Neuroimage; 1999. p. 125.
8. Sladky R, Friston KJ, TrÃ¶stl J, Cunnington, R., Moser, E., & Windischberger, C. Slice-timing effects and their correction in functional MRI. 2011. p. 588-594.
9. Whitfield-Gabrieli S, Nieto-Castanon A, Ghosh S. Artifact detection tools (ART). Cambridge, MA2011. p. 11.
10. Power JD, Mitra A, Laumann TO, Snyder AZ, Schlaggar BL, Petersen SE. Methods to detect, characterize, and remove motion artifact in resting state fMRI. *Neuroimage*. Jan 01 2014;84:320-41. doi:10.1016/j.neuroimage.2013.08.048
11. Nieto-Castanon A. Preparing fMRI Data for Statistical Analysis. fMRI techniques and protocols: Springer; Submitted.
12. Calhoun VD, Wager TD, Krishnan A, et al. The impact of T1 versus EPI spatial normalization templates for fMRI data analyses. *Hum Brain Mapp*. Nov 2017;38(11):5331-5342. doi:10.1002/hbm.23737
13. Ashburner J. A fast diffeomorphic image registration algorithm. *Neuroimage*. Oct 15 2007;38(1):95-113. doi:10.1016/j.neuroimage.2007.07.007
14. Ashburner J, Friston KJ. Unified segmentation. *Neuroimage*. Jul 01 2005;26(3):839-51. doi:10.1016/j.neuroimage.2005.02.018
15. Nieto-Castanon A. FMRI denoising pipeline. Handbook of functional connectivity Magnetic Resonance Imaging methods in CONN: Hilbert Press 2020.
16. Friston KJ, Williams S, Howard R, Frackowiak RS, Turner R. Movement-related effects in fMRI time-series. *Magn Reson Med*. Mar 1996;35(3):346-55. doi:10.1002/mrm.1910350312
17. Hallquist MN, Hwang K, Luna B. The nuisance of nuisance regression: spectral misspecification in a common approach to resting-state fMRI preprocessing reintroduces noise and

obscures functional connectivity. *Neuroimage*. Nov 15 2013;82:208-25. doi:10.1016/j.neuroimage.2013.05.116
18. Behzadi Y, Restom K, Liau J, Liu TT. A component based noise correction method (CompCor) for BOLD and perfusion based fMRI. *Neuroimage*. Aug 01 2007;37(1):90-101. doi:10.1016/j.neuroimage.2007.04.042
19. Chai XJ, Castañón AN, Ongür D, Whitfield-Gabrieli S. Anticorrelations in resting state networks without global signal regression. *Neuroimage*. Jan 16 2012;59(2):1420-8. doi:10.1016/j.neuroimage.2011.08.048
20. Nieto-Castanon A. Functional Connectivity measures. Handbook of functional connectivity Magnetic Resonance Imaging methods in CONN: Hilbert Press; 2020. p. 26-62.
21. Nieto-Castanon A. Brain-wide connectome inferences using functional connectivity MultiVariate Pattern Analyses (fc-MVPA). *PLoS Comput Biol*. Nov 2022;18(11):e1010634. doi:10.1371/journal.pcbi.1010634
22. Nieto-Castanon A. General Linear Model. Handbook of functional connectivity Magnetic Resonance Imaging methods in CONN: Hilbert Press. p. 63-82.
23. Worsley KJ, Marrett S, Neelin P, Vandal AC, Friston KJ, Evans AC. A unified statistical approach for determining significant signals in images of cerebral activation. *Hum Brain Mapp*. 1996;4(1):58-73. doi:10.1002/(SICI)1097-0193(1996)4:1<58::AID-HBM4>3.0.CO;2-O
24. Nieto-Castanon A. Cluster-level inferences. Handbook of functional connectivity Magnetic Resonance Imaging methods in CONN: Hilbert Press; 2020. p. 83-104.
25. Chumbley J, Worsley K, Flandin G, Friston K. Topological FDR for neuroimaging. *Neuroimage*. Feb 15 2010;49(4):3057-64. doi:10.1016/j.neuroimage.2009.10.090
